# The Influence of Time, Atmosphere and Surface Roughness on the Interface Strength and Microstructure of AA6061–AA1050 Diffusion Bonded Components

**DOI:** 10.3390/ma16020769

**Published:** 2023-01-12

**Authors:** Michael Ben-Haroush, Brigit Mittelman, Roni Shneck, Elad Priel

**Affiliations:** 1 Department of Materials, Nuclear Research Center Negev (NRCN), Be’er-Sheva 84190, Israel; 2 Department of Materials Engineering, Ben-Gurion University of the Negev, Be’er-Sheva 84105, Israel; 3 Department of Mechanical Engineering, Center for Thermo-mechanics and Failure of Materials, Shamoon College of Engineering, Be’er-Sheva 84100, Israel

**Keywords:** solid-state diffusion bonding, bonding strength, Al alloys, oxide film, FE

## Abstract

Diffusion bonding experiments followed by tensile testing were conducted on cylindrical pairs of AA6061–AA1050 aluminum alloys. The influence of bonding time, atmosphere and surface roughness on the resulting interface strength was studied. Metallurgical characterization was performed to study the quality of the bonded interface for different process conditions, and also to investigate the process of oxide formation on the specimen surface. Finite element analysis of the bonding experiments was used to study the thermo-mechanical fields during the bonding process. Using a cohesive zone approach for modelling the bonded interface, the bond strength for the different process parameters was quantified. The results demonstrate that high bond strength can be obtained even for specimens bonded in an air furnace, provided the surface roughness is low. When the surface roughness increases, specimens bonded in air show a reduction in interface strength, which is not observed for specimens bonded in vacuum. Inspection of the bonded interface suggests that this reduction in interface strength can be attributed to oxidation and pockets of air trapped between the asperities of the contact surface, which hinder diffusion and plastic flow.

## 1. Introduction

Multi-layered composite components are engineering components that are comprised of several metallic layers that are bonded together to attain one sheet, plate, rod or tube [1,2,3]. Theoretically, it is possible to tailor the effective mechanical, thermal and physical properties of such components by choosing the optimal combination of constituents and layer thickness ratio or layer geometry for a given engineering application [4,5]. In many applications, in the automobile and aerospace industries, aluminum alloys are a prime candidate as the main constituent of the multi-layered composites due to the requirement of high effective strength-to-weight ratio. A critical parameter that is crucial to the applicability of layered composites is the interface strength between the different composite layers [6,7].

Solid-state diffusion bonding has become an attractive technique of joining the different composite layers due to the good quality that can be achieved with similar and dissimilar materials [8,9]. A solid-state welding process is one in which two nominally flat surfaces are joined at elevated temperatures under an external pressure for specific bonding times. When the external pressure results in significant plastic deformation of the materials, the process is termed bonding by plastic forming. Such processes include hot roll bonding, hot forge bonding and co-extrusion [2,8,10]. In bonding by plastic forming, the process time is short, but the final dimensions of the composite and the different layers are hard to control due to the plastic flow of the different martials [2]. If one wishes to control the final dimensions of the composite, and the thickness of the different layers of the composite, the external pressure is limited to not plastically deform the bulk of the material. Such processes are termed Diffusion Bonding (DB) [11]. The major advantages of DB relative to other conventional joining methods (such as welding) is that it allows lower bonding temperatures and the ability to join dissimilar materials with large differences in the physical properties [12,13]. 

**Figure 1:**
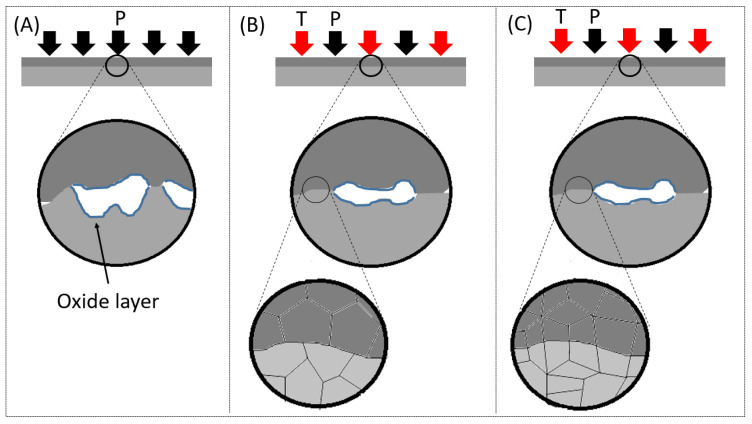
The three different stages of a diffusion bonding process: Initial static pressure (**A**), Heating of the component to the bonding temperature (**B**), Holding at the bonding temperature (**C**).

Diffusion bonding is usually conducted in several stages. The first stage is the initial contact of the joined surfaces under static pressure. The applied pressure results in local plastic deformation of the surface asperities on both sides of the interface. Plastic deformation of the surface asperities disrupts the oxide film and exposes a clean metallic surface and also produces intimate contact between the base metals. Surface finish will determine the height of the asperities present at the surface and control the real area of contact [9,14]. The second stage involves heating the components to the bonding temperature, which facilitates additional plastic deformation at the interface and increases metal-to-metal contact and interface void closures. Stage three involves holding the components at the bonding temperature to facilitate atomic inter-diffusion, recrystallization and/or grain growth across the bonded interface [14,15]. The three different stages in the diffusion bonding process are illustrated in Figure 1. 

Solid state bonding is formed by short range diffusion that is usually gradual and homogeneous, hence the mechanical properties and microstructure at the bonded region show better properties than joining by welding [15,16]. This approach avoids solidification and heat effects and the formation of intermetallic compounds, thus it may form satisfactory bond strength without significant deformation. However, there are process disadvantages as well: most of the processes require stringent cleaning of the interface surfaces as well as specific tooling and specialized equipment, and they are rarely portable. The bond interface has to be protected from oxidation in either vacuum or inert gas, and there are also difficulties with quality monitoring and inspection, and repairing of defective joints [14].

Diffusion bonding of aluminum alloys is greatly influenced by bonding conditions such as temperature, external pressure, holding time and surface roughness [14], but the major difficulty in the solid-state bonding of aluminum comes from its tenacious surface oxide film, which cannot be avoided under normal manufacturing conditions [3,6,17,18]. The oxide film prevents the close contact between base materials and acts as a diffusion barrier of alloying atoms, which results in poor bonding quality [19,20] and effective mechanical strength. The different process parameters that influence the bond strength of AA5083, AA6082 and AA7075 pairs was investigated in [21]. Bonding was performed in a vacuum chamber at temperatures of 490–520 °C, bonding pressure of 5–15 MPa and holding time of 15–45 (min). It is reported that the temperature has the greatest influence on the bonding strength, followed by the pressure and the holding time. Using tensile tests, a maximum bonding strength is reported for a bonding temperature of 520 °C, bonding pressure of 10 MPa and holding time of 30 (min). The reported bonding strength at these conditions is 31 MPa, 36 MPa and 36.9 MPa for AA5083, AA6082 and AA7075, respectively. Higher values are reported for the interface shear strength, but generally the bonding interface strength is much lower than the strength of the constituents that makeup the composite. The interface strength of dissimilar SSM7075- and SSM356-bonded pairs was investigated in [22]. Here too, the authors reported that the bonding temperature (in an Argon atmosphere) was the dominant factor governing the interface strength. Tensile testing resulted in a maximum average bond strength of 95 MPa for a bonding temperature of 500 °C and a holding time of 120 (min). The authors reported that the bond efficiency compared to the base material strength was only 56%. Diffusion bonding of dissimilar AA6061–AA7075 pairs at bonding temperatures of 325–425 °C (in a vacuum chamber), bonding pressure of 2–18 MPa and holding time of 15–75 min was investigated in [23]. Using ram tensile and lap shear specimen configurations, a maximum bonding strength of about 77 MPa in tension and 55 MPa in shear was obtained at a bonding temperature of 375 °C, a bonding pressure of 10 MPa and a holding time of 45 (min). These results seem to somewhat contradict the trends observed in the previous studies. For example, it is reported in [20] that, for the same temperature and holding time, an increase in bonding pressure resulted in lower interface bond strength. It is also reported that increasing the bonding temperature to 400 °C and the holding time to 60 (min) at the same bonding pressure also resulted in lower interface bond strength. To achieve a sound bond interface between aluminum alloys, it is necessary to remove the surface oxide or at least partially disrupt its continuity [7,24]. Niu et al. reported that in order to achieve high-quality joints by conventional diffusion bonding of Al, 1060 high temperature (above 500 °C) and large external pressure (more than 6 MPa) are required. In their research, they used Ar ion bombardment to remove the oxide film and to study the effect of surface modification such as roughness and structure on the diffusion bonding of Al/Al at lower temperature and pressure (450 °C and 5 MPa, respectively). Nevertheless, even following the surface treatment, the average interface strength is about 46 MPa, indicating a bond efficiency of only 60%. 

The present study focuses on determining the interface strength obtained by diffusion bonding of commercial aluminum alloy AA6061 to AA1050. The precipitation-hardenable AA6061 is favored for aerospace components and automotive parts and has superior strength relative to that of commercially pure aluminum [10,25]. On the other hand, pure aluminum is a soft, nonmagnetic, ductile metal that is known for its excellent corrosion resistance. Therefore, it would be beneficial to combine the good mechanical properties of 6061 Al and the good environmental resistance of pure Al [24].

From a review of the literature, it is evident that oxidation of the surface plays a critical role in the bond quality and the interface strength. Nevertheless, even when the DB process is conducted in a controlled environment, conflicting results regarding the interface strength of various aluminum pair bonding are reported. The purpose of this research is to investigate the influence of process atmosphere and its impact on the bonding quality. A particular interest was given to the effect of oxidization during the bonding process. The process parameters investigated are bonding time, atmosphere and surface roughness. Finite element analysis was used to examine the time dependent bonding pressure at the interface during the heating process. Computational models were also utilized to study the bi-material tensile tests utilizing a cohesive zone approach for the bonded region. 

The manuscript is structured as follows. In Section 2, the bonding experiments, tensile testing and material characterization are described. Section 3 is devoted to the computational modeling. The experimental results are described in Section 4 and discussed in Section 5. Summary and conclusions are given in Section 6. 

## 2. Materials and Methods

**Figure 2:**
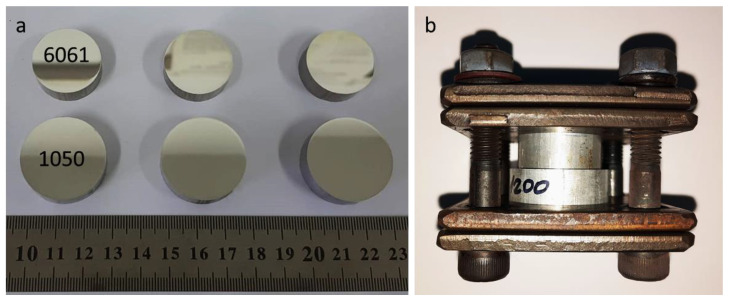
(**a**) Cylindrical AA1050 and AA6061 specimens after polishing; (**b**) Bonding couple before DB experiments.

Cylindrical-shaped specimens were cut and machined from a 30 mm diameter AA1050 rod and a 25 mm diameter AA6061 rod. The cylindrical specimens were cut to a 10 mm height and then mechanically polished by conventional grinding and polishing techniques to an ultimate 1 μm diamond finish in order to remove the oxide film and obtain a smooth surface. Then, the specimens were washed with running water and dried with pressurized air (Figure 2a). The specimens were placed one on top of another and clumped together in a small jig consisting of four steel plates connected by four 0.25″ screws. Each screw was fastened up to a torque of 25 Nm. The configuration of the specimens in the device is presented in Figure 2b.

In order to test the effect of atmospheric oxidization during the bonding process, the cylindrical specimens were polished, and similar specimen pairs were heated in two different ovens: a standard furnace under atmospheric pressure and a vacuum furnace under a vacuum of 1 × 10^−5^ mBar. 

### Bonding and Debonding Experimental Setup

The diffusion bonding couples were heated to 550 °C with a heating rate of 5 °C/min and held at this bonding temperature for 1, 3 and 24 h. To avoid thermal shock, the samples were cooled to room temperature in the work chamber. The temperature during the bonding process was measured by two K-thermocouples, which were kept close to the specimens. 

**Figure 3:**
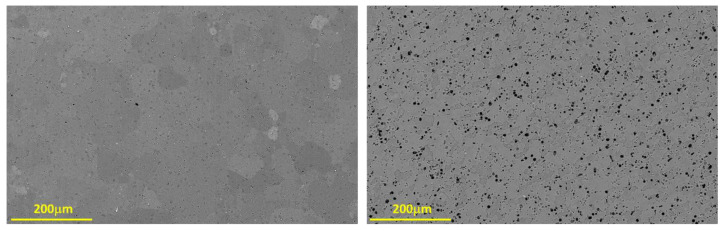
Microstructure of AA1050 (up) and AA6061 (down) specimens before DB experiments. (**Left**)—Optical microscope. (**Right**)—Electron microscope.

To examine the influence of surface roughness on bonding quality, the contact surfaces of the specimens were polished to three different polishing grades: P240 and P1200 grit SiC grinding papers, and 1 µm diamond pastes. Then, the diffusion bonding experiments were carried out at the same parameters mentioned above for a constant bonding time of 24 h. Table 1 lists the process parameters for each diffusion bonding experiment. The chemical composition of the aluminum alloys is given in Table 2 and the initial microstructure is shown in Figure 3. 

**Figure 4:**
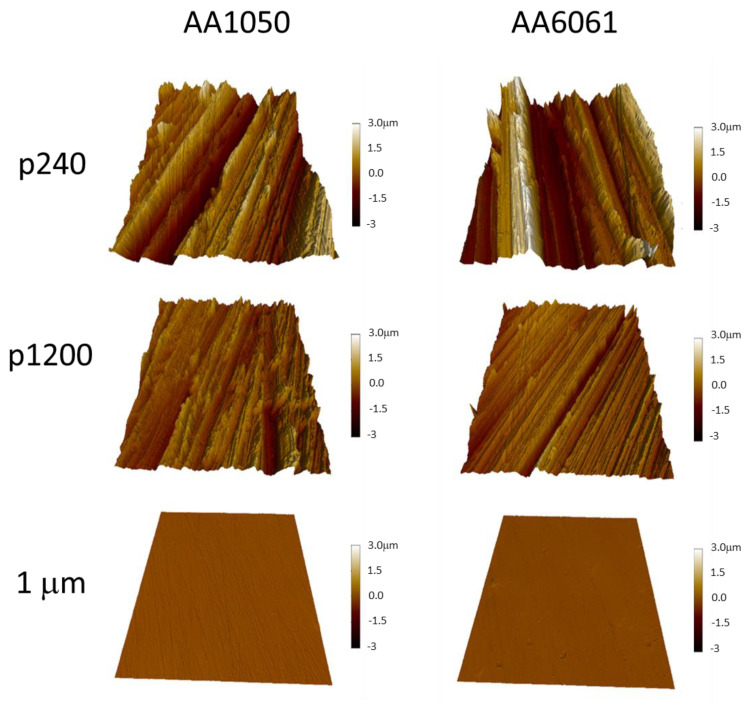
Surface topography of AA1050 and AA6061 specimens before DB experiments characterized by Atomic Force Microscope. The full color scale is 10 μm.

The surface roughness of the pre-bonding samples after polishing was measured by a 3D measuring laser confocal scanning microscope LEXT OLS 5000, and the topography was characterized by an Atomic Force Microscope (AFM) (Figure 4). The results are shown in Table 3; they are based on an average of 3 measurements performed on representative samples. As reported in [26], we used the Ra (µm) parameter to describe the surface roughness. Ra is the most commonly used parameter for assessing the smoothness of materials and is an arithmetic mean of the vertical departures of the roughness profile from the mean line. 

**Figure 5:**
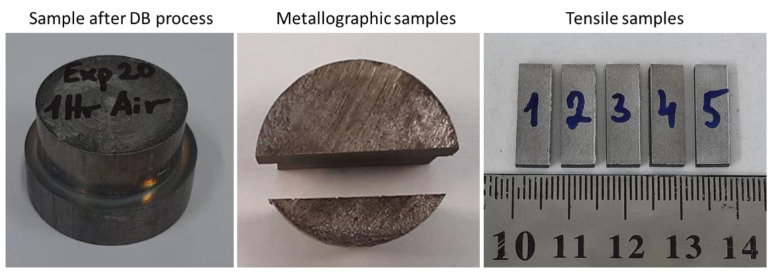
Samples after DB experiment and EDM cut for tensile and metallographic characterizations.

The bond quality of joints was assessed through examination of the interface microstructure and by measurement of the bond strength. Following the bonding experiments, the specimens were sectioned perpendicularly to the bond line by an Electric Discharge Machine (EDM) into tensile and metallographic specimens, as displayed in Figure 5. 

The interface microstructure was analyzed by Scanning Electron Microscope (SEM, Phenom, Oberkochen, Germany) equipped with an Energy Dispersive Spectroscope (EDS). In addition, some characterization was conducted using high resolution scanning electron microscope (using a Thermo-Fisher Verios 460 L field-emission SEM, Waltham, MA, USA). Each sample was prepared using standard metallographic technique, and Keller’s reagent was used for etching. Hardness measurements were carried out before and after the diffusion bonding processes. An indention test (Zwick Roell indentec Machine, Ulm, Germany) was used to measure the hardness of the parent materials, with a 3 Kg testing force and 10 s dwell time. A total of 10 measurements at each side across the bulk materials (AA1050 and AA6061) were taken, and the results were averaged. 

The debonding tensile tests were conducted using a 10 kN Shimadzu electro-mechanical test machine. All tension experiments were conducted at room temperature until failure with a loading rate of 2 mm/min. For each bonding pair, 5 tensile specimens were tested, and the average values are reported. 

**Figure 6:**
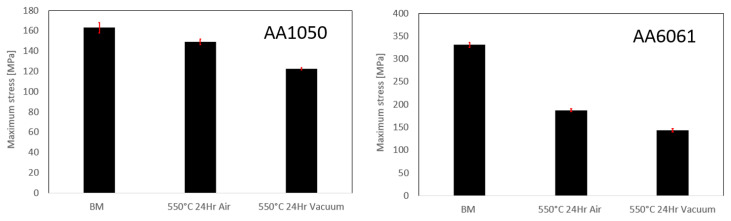
Tensile strength of AA1050 and AA6061 before and after heat treatment for 24 h at 550 °C.

For a qualitative comparison between the tensile properties of the parent material and the bond strength measured, specimens from bulk AA1050 and bulk AA6061 were also tested using the same tension test specimen configuration shown in Figure 4. These specimens were designated “BM” after heating to 550 °C for 24 h with a heating rate of 5 °C/min. The tensile properties of the base material before and after heat treatment at both air and vacuum atmosphere are shown in Figure 6. Both materials undergo annealing during the heat treatment, and their strength decreases with the annealing time. The differences in the parent material tensile strength between the air and vacuum furnaces are the result of differences in the cooling times, which lead to differences in average grain size, as explained in Appendix A.

## 3. Computational Modeling

**Figure 7:**
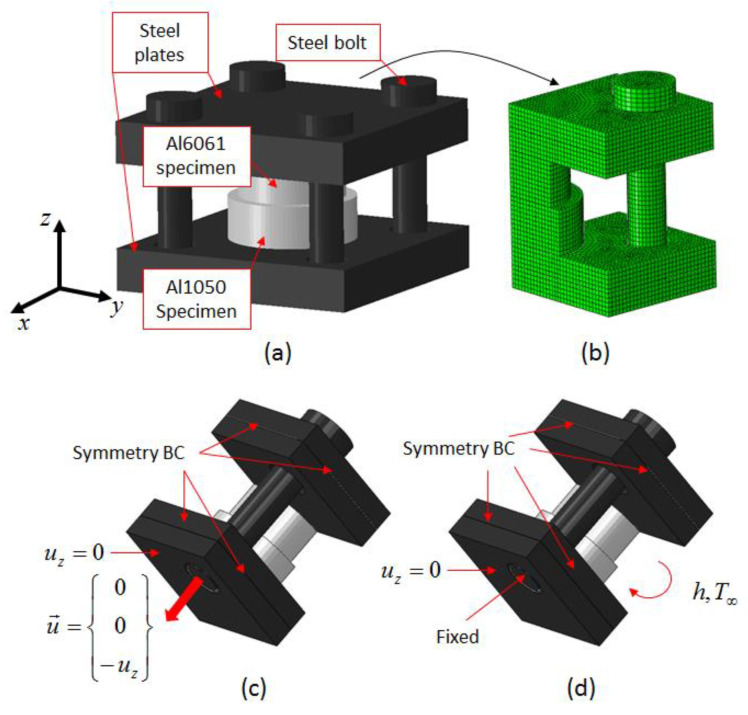
Finite element model of the bonding system: (**a**) Model geometry (mirrored to show the whole system), (**b**) Representative mesh, (**c**) bolt tightening stage, (**d**) heating stage.

The diffusion bonding process is governed by local temperature and pressure at the interface between the specimens. To gain understanding of the thermo-mechanical fields that develop at the interface during the bonding process, a finite element model of the system shown in Figure 2b was developed using the commercial code ABAQUS [27]. The model was used to simulate the initial bolt tightening stage, followed by heating in the furnace to model the bonding stage. In Figure 7, the geometric model (taking into account the system symmetry) and a representative mesh are shown. 

The computational mesh was constructed using linear 8 node brick elements with both displacement and temperature degrees of freedom (termed C3D8T elements in ABAQUS). Full integration was used with an implicit time integration scheme utilizing the automatic time step option. Contact was enforced between all the different parts of the system using the penalty method with a constant coulomb friction coefficient of μ = 0.3 [7]. Hook’s law was used to model the material in the elastic region, while J2 plasticity with isotropic strain hardening was used for the elasto-plastic range. The elastic and thermal material properties for the different materials are taken from the literature [28,29] and provided in Table 4.

The elastic modulus was taken as temperature dependent, but the thermal properties were kept constant as initial computations showed that temperature dependent thermals result in a negligible difference in the results. The flow stress as a function of temperature for the Al6061 was taken from the literature [26], while the flow stress for the Al1050 were characterized previously by the authors in [30]. It was assumed that the steel deforms only in the elastic range, even at 550 °C. For the initial stage of the computation, displacement boundary conditions were applied to the bolt. The axial displacement was determined using the computed axial force in the bolt following standard analytical calculations of bolt tightening using the applied experimental torque of 25 Nm [31]. For the second stage of the computation, the initial temperature of the system was set to T = 25 °C with convection boundary conditions h = 100 (W/m^2^C), T = 550 °C applied to all exposed surfaces. 

**Figure 8:**
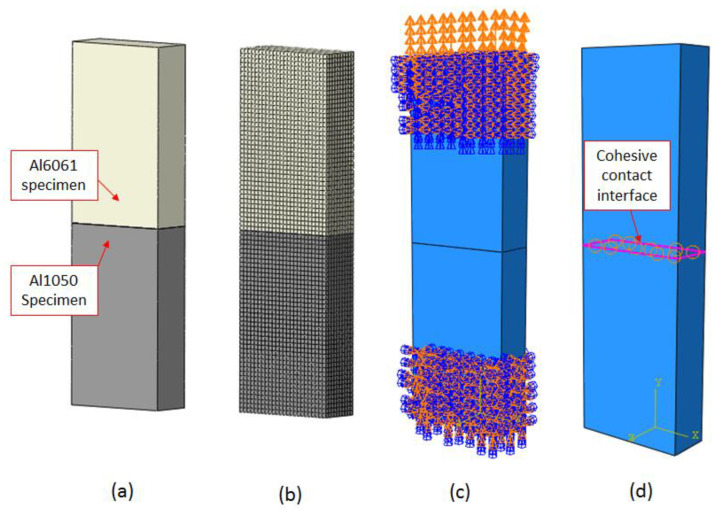
Computational model of the tensile tests: (**a**) Model geometry; (**b**) Representative mesh; (**c**) Boundary conditions; (**d**) cohesive contact interface for modelling bonded region.

The tensile specimens extracted from the bonded specimen were not conventional. To study the mechanical fields that develop in the specimen, an additional computational model was developed for simulation of the tensile tests. The geometry, representative mesh and boundary conditions for these models are given in Figure 8.

**Figure 9:**
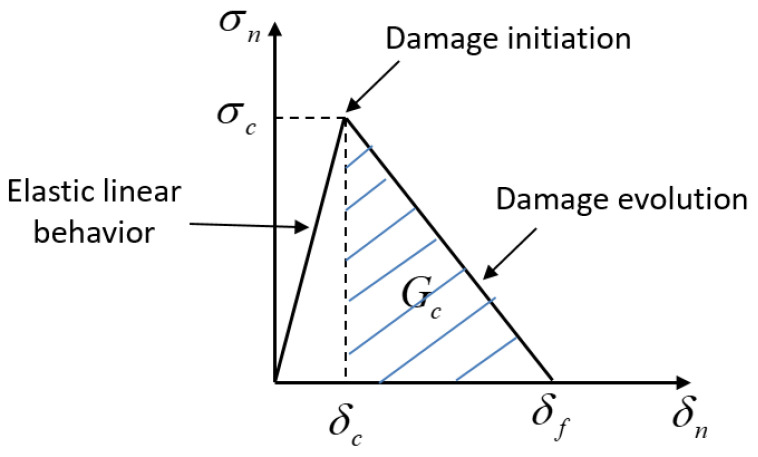
Bi-linear traction separation function for modeling damage at the bonded interface.

The tensile specimen models were meshed using eight nodded linear brick elements (termed C3D8 in ABAQUS) with full integration. To model the bonded interface between the Al1050 and Al6061, a cohesive contact model was utilized [32]. The bi-linear traction separation function shown in Figure 9 was utilized in the interface. The cohesive contact model assumes that, for each point across the interface, the stress-displacement response is linear elastic until a critical interface stress value, $\sigma_{c},\;$is reached. Once this critical stress is reached, the local stiffness begins to degrade. From a micromechanical point of view, this reduction in stiffness is attributed to small voids that grow along the interface. Once a critical displacement between the interface surfaces is reached, at a given point, that particular point can no longer transfer load between the bonded surfaces and is assumed to be de-bonded. The area under this damage evolution curve is defined as the critical energy density per unit area, $G_{c}$, as illustrated in Figure 9. 

The elastic behavior of the bonded interface was assumed to be similar to the bulk aluminum response. This assumption is implemented in the finite element program by the default contact enforcement method option, which sets the interface stiffness value to be the average stiffness calculated from the elastic parameters of the material on both sides of the interface. The critical values for damage initiation, $\sigma_{c}$, and critical energy density, $G_{c}$, were obtained from comparison of the simulation results to the measured experimental values. Following the bonding experiments, no notable change in specimen shape or size was measured. Therefore, it was assumed that no significant residual stress fields from the bonding process exist in the tensile specimens. It was also assumed that the specimen cutting process did not damage the bonded interface. The flow stress of the material in the bonding models was characterized using tension tests of the same geometry presented in Figure 7, of a single material. The resulting stress–strain relations are provided in Table 5, in the form of the Ludwig relation: $\sigma =K\cdot \varepsilon^{n}+\sigma_{0}$.

**Figure 10:**
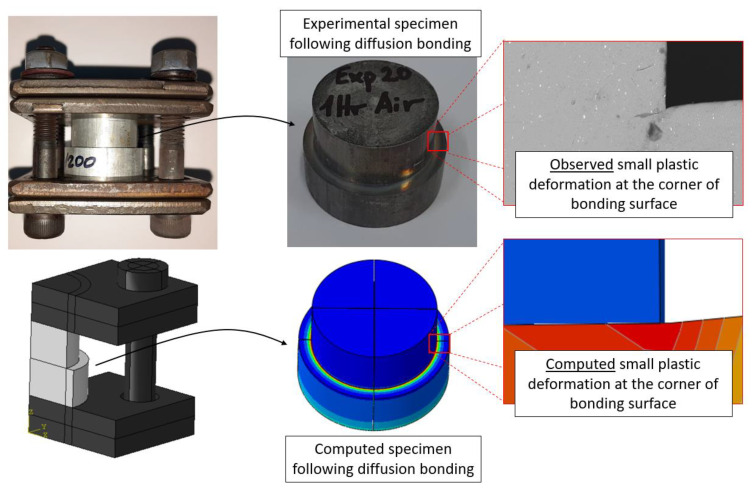
Computed vs. Observed local deformation at the bonding interface corner following the diffusion bonding process.

All computational models used in this study underwent convergence tests for solution verification, as discussed in prior publications of the authors [27]. Validation of the computed temperature for the bonding stage was conducted by comparing the computed time dependent temperature of the system to values measured at different times during the DB process. Validation of the computed deformation fields was conducted by comparing the observed and computed local deformation of the specimens at the corners of the interface surface. An example of this comparison can be seen in Figure 10.

## 4. Results

### 4.1. Time and Atmosphere Effects on Bonding Strength

**Figure 11:**
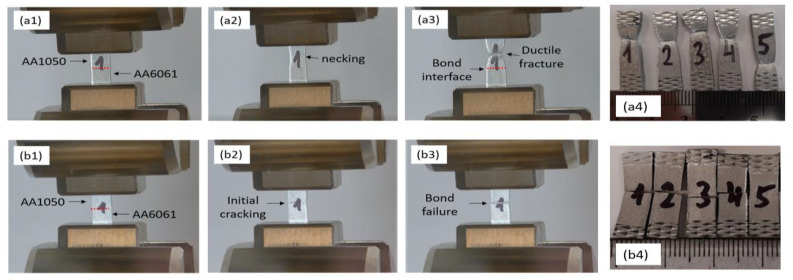
The two types of failure modes observed in the tensile tests: Ductile failure of the AA1050 region (**a1**–**a4**) and brittle failure of the bonded interface (**b1**–**b4**). These modes appear consistently in 5 repetitions of the test (**a4** and **b4**).

Two types of failure modes were observed upon the DB of specimens with the best surface quality in air and in vacuum furnaces: failure of the bonded interface or ductile failure of the AA1050 region of the bonded specimen, as shown in Figure 11. Table 6 summarizes the failure location of the tensile specimens. 

**Figure 12:**
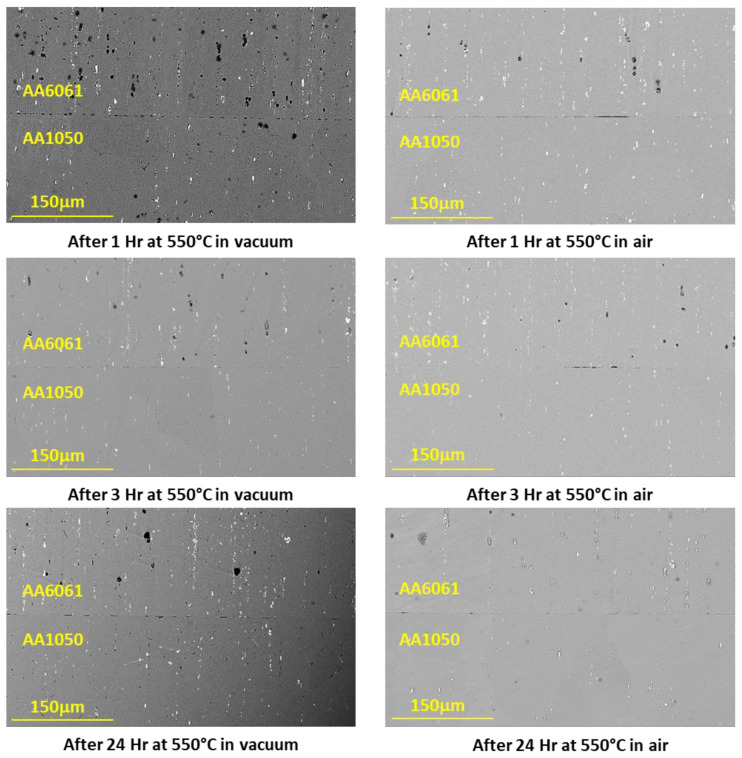
Typical interface of AA6061–AA1050 pairs after diffusion bonding experiment for different holding times and atmosphere.

Figure 12 shows backscattered electron images of the AA6061–AA1050 interface for the first set of experiments. After an hour, many discontinuities along the interface are seen with some areas where material continuity exists. As the duration of the diffusion process increases, the continuous regions across the interface expand.

**Figure 13:**
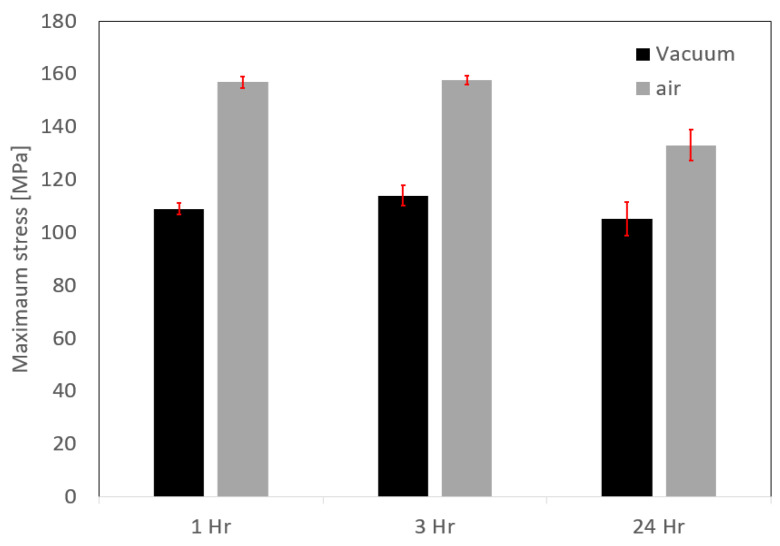
Tensile strength of 6061–1050 pairs after DB for different holding times and atmosphere.

The tensile strength of the AA1050-AA60601 pairs obtained after DB at 550 °C for 1, 3 and 24 h in vacuum and air are shown in Figure 13. After a slight increase in strength after 3 h DB treatment, a small decease of strength occurs after 24 h. Because most of the specimens failed in the bulk the AA1050 region, this decrease in strength reflects the properties of the annealed material, while the interfaces exhibit higher tensile strength than the bulk AA1050 (excluding 24 h in air). It is also interesting that the ultimate stress of the specimens bonded in air is higher compared to those bonded in vacuum due to the shorter annealing time in the air furnace, as shown previously in Figure 6.

**Figure 14:**
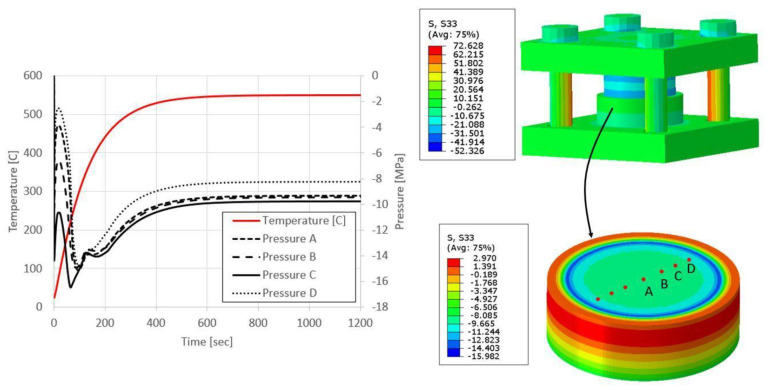
Interface temperature and pressure at different locations (A–D) as a function of time during the bonding process.

As stated in the introduction, the interface pressure can also influence the bonding quality. To estimate the interface pressure, the thermo-mechanical finite element model described in Section 3 was applied. In Figure 14, the time dependent pressure and temperature fields at different location along the interface are presented. It can be seen that the interface pressure is not constant during the bonding process. An initial pressure is obtained following the tightening of the bolt. During the heating process, when the temperature is increased, further high pressure develops due to the differences between the thermal expansion coefficients between the aluminum and the steel. A maximal pressure of 16 MPa develops during the early stages of the bonding process, when some temperature gradient exists between the aluminum and steel parts of the bonding system (see Figure 1B). These high pressures drop to 8–10 MPa only when the steady state temperature is reached. These values are in the same range as reported in previous studies [18,19,20]. Figure 14 shows that the pressure variation across the contact surface is small. 

### 4.2. Surface Roughness Effects

In this section, the effect of surface roughness on bond quality and strength is presented. Table 7 summarizes the failure location of the tensile specimens in all bonding pairs in the second set of experiments.

**Figure 15:**
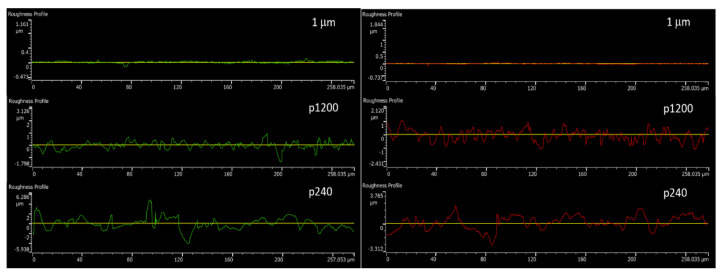
Surface roughness of AA1050 (**right**) and AA6061 (**left**) samples after polishing to different levels.

**Figure 16:**
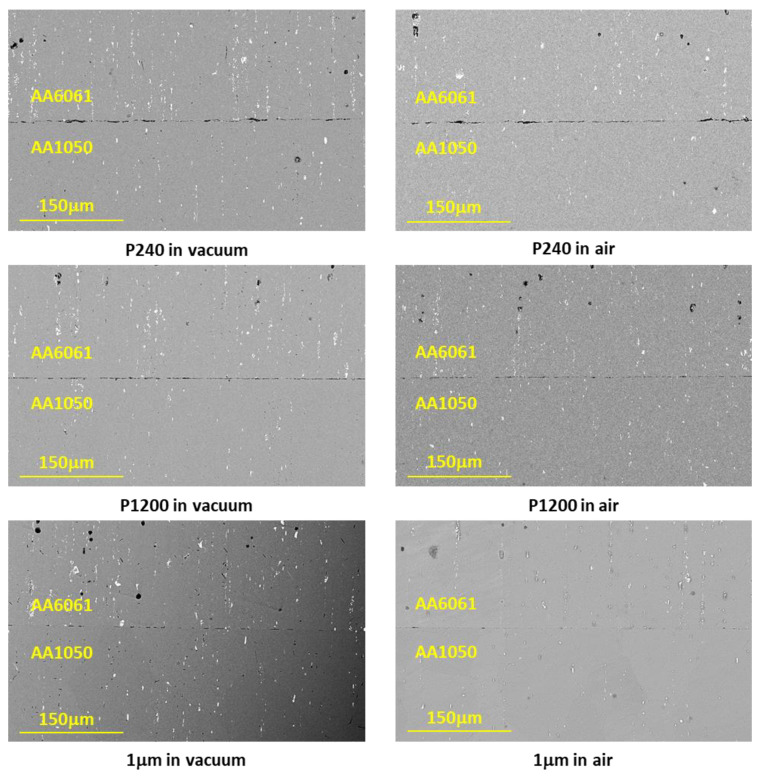
Typical interface of AA6061–AA1050 pairs (different surface roughness) after DB experiments for 24 h in vacuum and in air.

Figure 15 shows the variation of AA6061 and AA1050 surface roughness using different polishing grits. Figure 16 shows the microstructure variations at the bonding interface for the different initial surface roughnesses with similar temperature and holding time. The continuity of the bonded interface increases with the decreasing of the initial surface roughness. 

**Figure 17:**
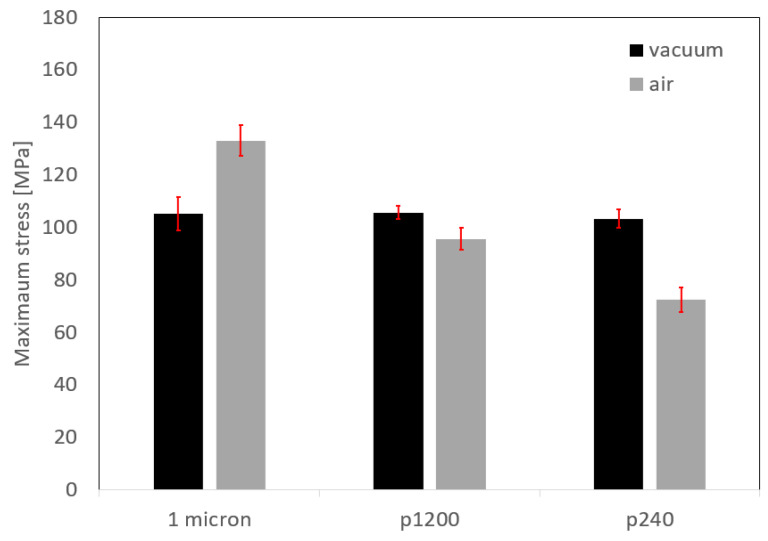
Tensile strength of AA6061–AA1050 pairs with different initial surface roughness after diffusion bonding experiments for 24 h in a vacuum and in air.

Figure 17 presents the interface strength for pairs with different initial surface roughness. All the specimens bonded in the air furnace failed at the bonded interface, and their strength declines as the surface roughness increases. The strength of the specimens bonded in vacuum is indifferent to the surface roughness. It is also apparent that the bond strength for specimens bonded in vacuum is close to the strength of the annealed AA1050 alloy (Figure 6). 

**Figure 18:**
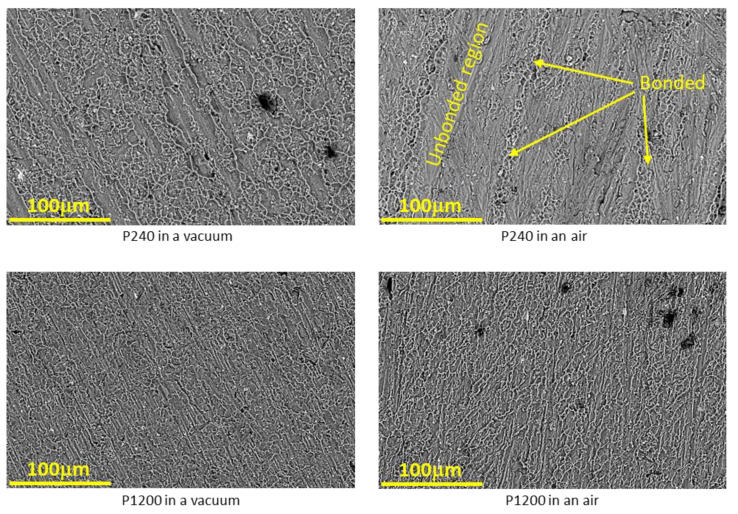
Fracture topography after tensile test of AA6061–AA1050 pairs with different surface roughness, also showing bonded and unbonded regions on the fracture surface.

All the tensile specimens of this second set of experiments failed, at least partially, at the interface. Only the specimens with 1 μm finish bonded in a vacuum furnace failed in the bulk material. The fracture surface morphology of the p240 and p1200 polished specimens was examined and is presented in Figure 18. The fracture at the interface is characterized by two kinds of morphologies: dimples, which indicate bonded areas and area which resemble the original surface after polishing. There is a difference in the amount and size of the dimples. For specimens bonded in a vacuum furnace, more prominent dimples are observed in the fracture surfaces of the specimens with a p240 finish, compared to specimens with p1200. On the other hand, for specimens that were bonded in the air furnace, the opposite trend is observed: more prominent dimples are observed in the fracture surfaces of the specimens with a p1200 finish. 

### 4.3. Bonding Strength 

**Figure 19:**
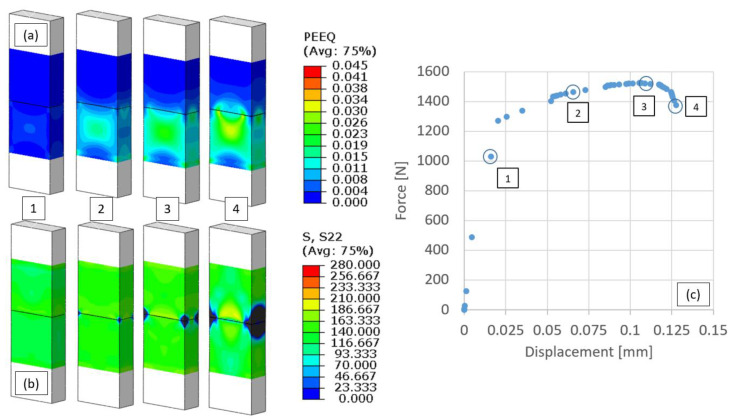
Computed evolution of the equivalent plastic strain (**a**), axial stress component (**b**) and the force displacement curve (**c**) at different stages of the tension test (points 1–4).

The numerical models of the tension tests of the diffusion bonded specimens (described in Section 3) were used to characterize the interface strength. In Figure 19, an example of the stress and strain fields that develop during the bi-material tensile tests is shown. The computed results show that, during the tensile test, the AA1050 region undergoes extensive plastic deformation compared to the AA6061. Stress concentrations along the edges of the bonding interface are evident. 

**Figure 20:**
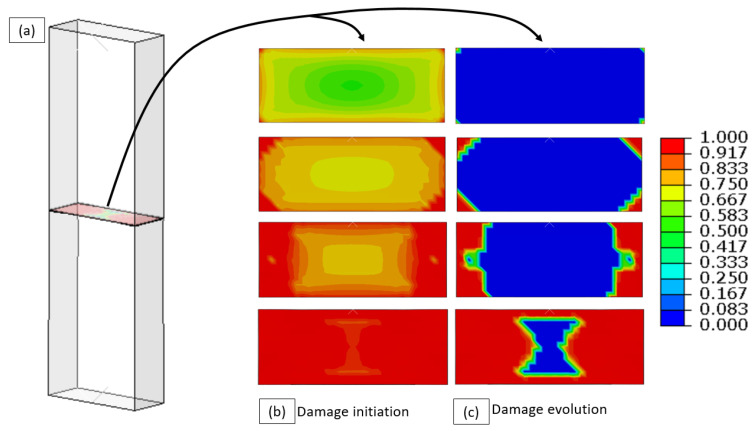
(**a**) The bonded surface in the computation bi-material specimen, (**b**) evolution of the damage initiation parameter and (**c**) of the damage evolution parameter.

In Figure 20, an example of the computed interface damage initiation and evolution at the bonded surface is shown. The parameter for damage initiation shown in Figure 20b defines the ratio between the local normal stress value at the interface and $\sigma_{c}$. A value of 1 indicates that the critical stress was reached, and local damage starts to accumulate. The parameter for damage evolution shown in Figure 20c defines the ratio between the local energy density and the critical energy density,$\;G_{c}$. In this case, a value of 1 indicates that local de-bonding has occurred. The de-lamination of the interface starts at the specimen corners and evolves correspondingly from the specimen corner, along the short edges and into the center of the bonding interface. This process of specimen de-bonding was also observed in the tensile experiments (see for example Figure 11(b2)). 

In cases where the failure from experiments was located at the interface, different cohesive strength values were guessed until the force at failure matched the average value for this group of experiments. In addition to the surface strength definitions within the model at the cohesive contact interface, a fracture energy value of $G_{c}=$ 2 × 10^−5^ (mJ/mm^2^) was chosen. In addition, an artificial viscosity coefficient of 5 × 10^−4^ was defined in order to ensure numerical stability. It was verified that the artificial viscosity does not influence the computed results. The interface strength, $\sigma_{c}$, deduced from the numerical models is presented in Table 8. One must not confuse the experimental value of UTS (shown in Figure 16) with the critical interface strength, $\sigma_{c}$, identified from the cohesive contact model. The former is a global value identified from the engineering stress–strain experimental curve and cannot be used to model interface failure, while the latter is a local property of the interface determined from the computational analysis, which also takes into account the deformation of the bonded region. The diffusion pairs treated in vacuum are found to have a stronger interface bonding compared to the ones treated in air. In addition, as expected, the smoother the interface of the diffusion pairs, the better the resulting interface strength. In the vacuum bonded pair with 1 μm roughness, the interface strength is higher than the UTS of the base metal, which is about 140 (MPa), because the failure is located at the base (AA1050) metal.

### 4.4. Microstructure of the Bonding Interface

**Figure 21:**
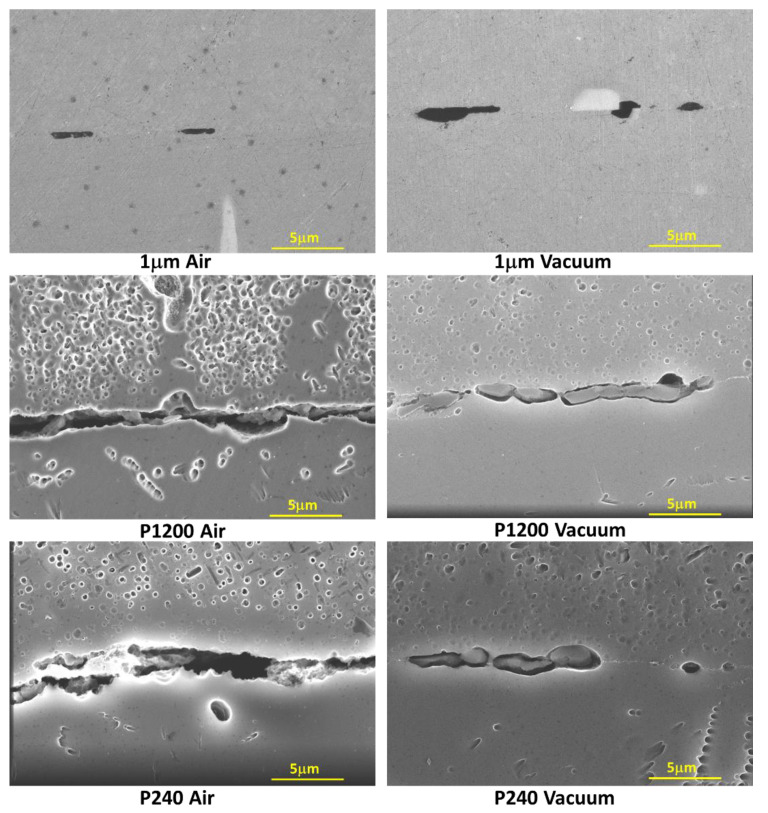
HRSEM microscopic characterization of the interface after DB of AA6061–AA1050 couples with different surface roughness in a vacuum and air oven.

From the microscopic results obtained for couples with different initial surface roughness (Figure 16), it can clearly be seen how the continuity at the interface increases with the quality of the initial surface roughness. Therefore, one would expect to see dependence between interface strength and interface continuity, and that the change in the interface strength trend will be similar for couples that have been in the different environments (vacuum/air), but this is not the case. The experimental strength results shows that the surface roughness has a significant effect on couples that underwent diffusion bonding in air when the interface strength decreases from 133 MPa (for 1 μm finish) to 72 MPa (for P240 finish). On the other hand, for couples that have been bonded in vacuum, it was found that the surface roughness has almost no effect on the interface strength and it remains almost constant (about 105 MPa). These results suggest that there may be a difference between the nature of the interface/oxide layer obtained between pairs that have been bonded in an air/vacuum environment. In order to test this hypothesis, focus was placed on characterizing the interface using HRSEM after polishing the surface (without etching) and examining the interface area. The results are shown in Figure 21 and show that indeed there is a difference between the interface obtained in a vacuum/air condition. 

**Figure 22:**
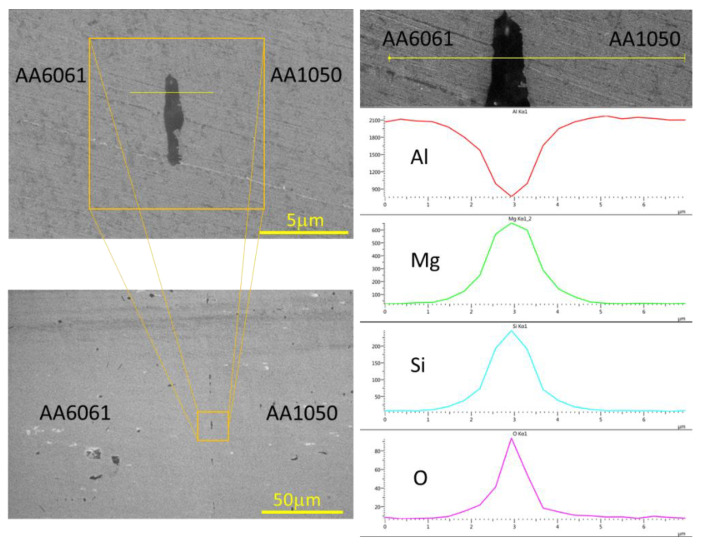
HRSEM EDS Line scan after 24 h DB in a vacuum oven of AA6061–AA1050 couples.

For AA6061–AA1050 couples that were bonded in vacuum, one can see a discontinuous oxide layer along the interface, in all the attached samples and for all the various initial surface roughnesses. On the other hand, for diffusion bonding performed in air, it can be seen that for a rough surface there is no oxide at the interface and there is a hole that indicates that the oxide that was there has been detached from the specimen during the polishing process. EDS line scans were performed to characterize the oxide at the interface; the results are shown in Figure 22. From the EDS results, it can be said that the oxide at the interface mostly consists of Mg, Si, O in varying compositions, and sometimes Fe as well (the white spots). The weight concentration of the different elements varied significantly at different locations for the same interface. As a consequence, a clear relation between the bonding environment and the resulting composition of the oxide layer could not be determined. 

## 5. Discussion

The effects of oxidation on the interface strength are manifested both for couples with a smooth surface that underwent diffusion bonding for different times in air and also for couples with different surface roughness that were treated in an air environment for 24 h. Where the surface is smooth, there is good contact between the surfaces of the samples (AA6061–AA1050), which makes it difficult for the oxygen to penetrate the interface, thus slowing down the oxidation rate. As evidence, it can be seen that after diffusion bonding for 1 and 3 h, the failure of the tensile samples was in the bonded material (AA1050 bulk) and not in the interface. On the other hand, after 24 h of oxidation in air, the failure is in the bonded interface, indicating penetration of the oxidation throughout the interface. In the second case of couples with different initial surface roughness that underwent diffusion bonding in an air furnace for 24 h, it can be seen that the rougher the surface, the more the strength of the interface decreases. This can be attributed to the oxidation of the interface because this trend does not exist for similar couples, which were in a vacuum furnace and had the same surface roughness. Thus, the detrimental effect of oxidation on the strength is subtle.

**Figure 23:**
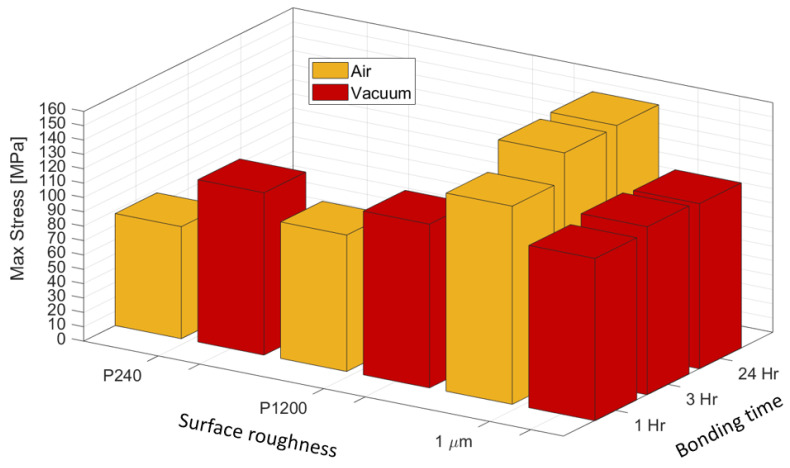
Summary of the bond strength as a function of time of DB treatment and surface roughness.

Figure 23 is a 3D plot that summarizes the bond strength as a function of time and surface roughness. It shows the surprisingly highest strength of air bonded joints of high-quality surfaces for short DB times and indifference of the strength of vacuum bonded joints with variation of roughness. 

The specimen with the highest strength failed in the AA1050 region and not at the bonding surface, indicating that the bond is stronger than the AA1050. The AA1050 region retained a relatively high strength because it was exposed to the shortest annealing treatment in the air furnace.

The DB involves two parallel processes: diffusion and plastic flow at asperities, which is probably more significant in rough surfaces. When the surface roughness is low, the contact pressure is sufficient to flatten the surface asperities, fracture any initial surface oxidation and provide material-to-material contact, which is required for inter-diffusion (Figure 16). Oxidation inhibits both these processes, therefore the closed air pockets and the relatively fast oxidation during DB in air interfere with the diffusion and the plastic deformation and result in reduced strength with increasing roughness. On the other hand, the limited oxidation and air pockets typical to DB in vacuum allowed diffusion and plastic flow to take place, which overwhelmed the fewer initial contact areas between rough surfaces and resulted in similar strengths of surfaces with different roughness. Despite the fact that the vacuum level was not sufficient to prevent oxidation of Al, Mg and Si (Figure 21 and Figure 22), the vacuum was enough to slow down the oxidation.

## 6. Summary and Conclusions

Diffusion bonding experiments followed by tensile testing were conducted on pairs of AA1050 and AA6061 cylindrical specimens in order to investigate the influence of bonding time, atmosphere and surface roughness on the bonded interface strength. Metallurgical characterization was utilized to study the quality of the bonded interface for the different process conditions and also to investigate the process of oxide formation on the specimen surface. Finite element analysis was utilized in order to compute the time dependent bonding pressure and the mechanical fields that develop during the bi-material specimen tensile tests. Using a cohesive zone approach, for modelling the bonded interface, the bond strength for the different process conditions was quantified. The results demonstrate that high values of interface bond strength can be obtained even for specimens bonded in an air furnace provided the surface roughness is low. Interface strength values of 100–130 MPa were obtained for the different DB process conditions for which failure occurred at the bonded interface. For specimens in which failure occurred in the AA1050 region of the bi-material specimens, the minimum average value of interface strength is taken as the AA1050 material strength for the corresponding heat treatment. When the surface roughness increases, the interface strength of specimens bonded in air decreases. The reduction in interface strength of the specimens with higher surface roughness bonded in air is attributed to pockets of air trapped between the asperities of the contact surfaces, which hinder diffusion during the dwell time in the furnace. The interface strength of specimens bonded in vacuum is not influenced by the surface roughness.

## Figures and Tables

**Table 1:** Diffusion bonding parameters used to bond AA1050 to AA6061.

Specimen No.	Time (Hr)	Atmosphere (mBar)	Polishing Level
1	1	8·10^−5^	1 μm
2	3		
3	24		
4	1	1013	
5	3		
6	24		
7	24	8·10^−5^	1200 grit
8			240 grit
9		1013	1200 grit
10			240 grit

**Table 2:** Chemical composition of AA1050 and AA6061 aluminum alloys in wt%.

	AA1050	AA6061
Element	wt%	wt%
Mg	0.0045	0.4844
Si	0.1712	0.3968
Cr	0.0015	0.0022
Mn	0.0012	0.0043
Fe	0.2123	0.1493
Cu	0.0005	0.0056
Zn	0.0023	0.0033
Zr	0.0017	0.0017
Ti	0.0088	0.0087
Al	balance	balance

**Table 3:** Surface roughness Ra of the pre-bonding samples after polishing to different polishing levels.

Alloy	Polishing Level	Ra (nm)
6061	1 μm	28.0
	P1200	305.0
	P240	1165.0
1050	1 μm	24.3
	P1200	395.0
	P240	792.0

**Table 4:** Elastic and thermal properties used in the thermo-mechanical simulation.

Material	E (GPa)	v	k (W/mC)	Cp (J/kgC)	α (1/C)
Al1050	30–70	0.33	200	860	2.4 × 10^−6^
Al6061	30–70	0.33	200	860	2.4 × 10^−6^
Steel	200	0.29	54	460	1.2 × 10^−6^

**Table 5:** The flow stress relation characterized from tension tests of the heat-treated materials.

Material	Al1050 BM	Al1050 24 h Air	Al1050 24 h Vacuum	Al6061 BM	Al6061 24 h Air
$\sigma_{0}\;\left(MPa\right)$	90	65	30	80	100
K (MPa)	300	240	165	1100	380
n	0.55	0.4	0.18	0.7	0.6

**Table 6:** Failure location of the tensile specimens for the first set of experiments.

Atmosphere &	Surface	
DB Time	Roughness	Failure Location
1 Hr Vacuum	1 μm	AA1050-Bulk
3 Hr Vacuum	1 μm	AA1050-Bulk
24 h Vacuum	1 μm	AA1050-Bulk
1 Hr Air	1 μm	AA1050-Bulk
3 Hr Air	1 μm	AA1050-Bulk
24 h Air	1 μm	Interface

**Table 7:** Failure location of the tensile specimens in the second set of experiments.

Atmosphere &	Surface	Failure
DB Time	Roughness	Location
24 h Vacuum	1 μm	AA1050-BM
24 h Vacuum	P1200	80%-AA1050-Bulk, 20% Interface
24 h Vacuum	P240	20%-AA1050-Bulk, 80% Interface
24 h Air	1 μm	Interface
24 h Air	P1200	Interface
24 h Air	P240	Interface

**Table 8:** Interface strength characterized from the numerical models.

Atmosphere & DB Time	Surface Roughness	$\sigma_{c}$ (MPa) (Damage Initiation)	Average Force at Failure from Experiments (N)
24 h Vacuum	1 μm	>140	1620
24 h Vacuum	P1200	130	1520
24 h Vacuum	P240	126	1480
24 h Air	1 μm	134	2100
24 h Air	P1200	120	1400
24 h Air	P240	100	1070

**Figure A1:**
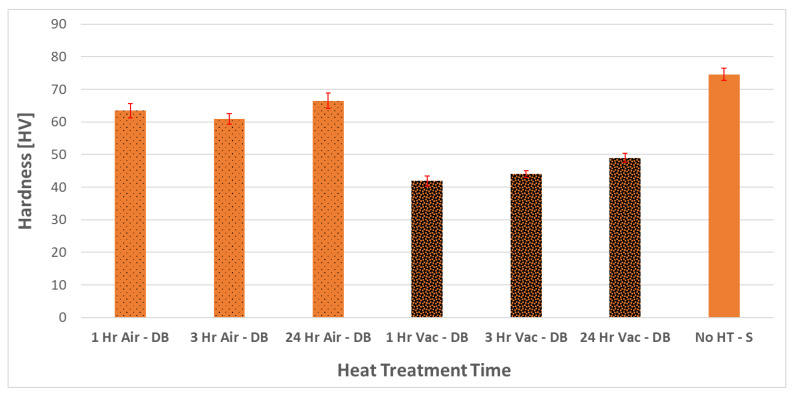
AA1050 hardness before and after DB process.

**Figure A2:**
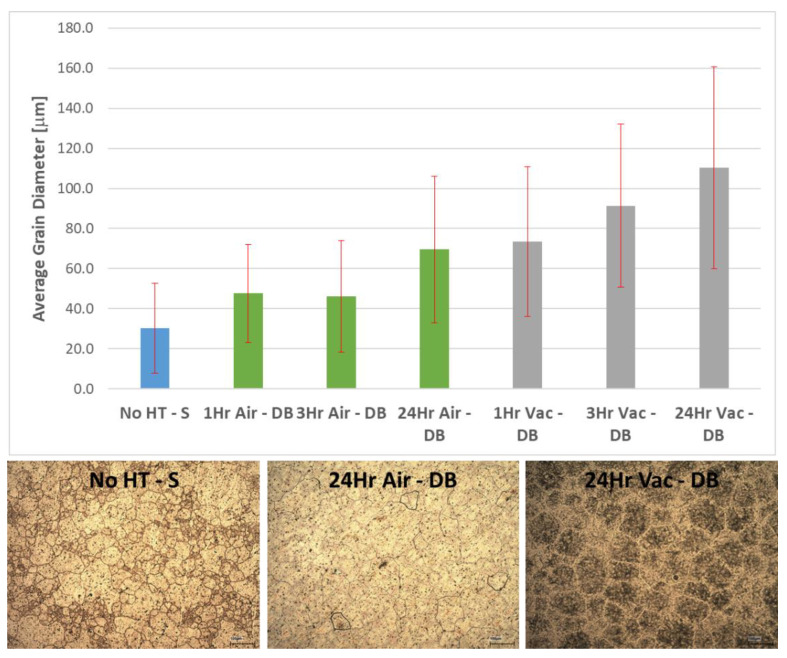
AA1050 average grain size distribution results before and after DB processes.

## Data Availability

The data presented in this study is available upon request from the corresponding author.

## Appendix A. Influence of Thermal Treatment on Bulk Material Strength

It is known from the literature that diffusion bonding in a vacuum oven can affect the strength of the base metal by the excessive thermal radiation due to the long period process of heating and cooling [15].

Table A1 lists the heating and cooling profiles in the two different ovens. The difference is mainly expressed in the cooling profile to room temperature, when the duration of cooling in the vacuum furnace lasts about 11.5 h longer than that obtained of the air furnace.

**Table A1:** Heat treatment profile.

	Heating Time	Duration Time at 550 °C	Cooling Time to 25 °C
Oven Type	(Hr)	(Hr)	(Hr)
Vacuum Oven	2:10	24:00	12:30
Air Oven	1:50	24:00	0:50

In order to examine the changes that occurred following the thermal treatments, hardness tests and measurements of the average grain size were performed before and after the thermal treatments. Because the failure of the DB tensile samples was always at the interface or at the AA1050 bulk, focus was placed on measuring the hardness and average grain size of the AA1050.

The hardness results (shown in Figure A1) of the AA1050 bulk material before heat treatment (marked as “S”) and after different time of DB (marked as “DB”) are in line with the findings obtained in the tensile tests. The measured hardness of the materials obtained after thermal treatment in an air furnace is greater than that obtained in a vacuum furnace. It can be seen that the hardness of the AA1050 before heat treatment is 74.6 Hv, and after DB for 24 h in air it is reduced to about 66.5 Hv. The same heat treatment conducted in a vacuum furnace resulted in a reduction in hardness to about 49 Hv.

When examining the variation in grain size as a function of the duration of the thermal treatment, a clear trend of grain size growth can be seen. The AA1050 average grain size distribution results (Figure A2) show that the initial grain size before thermal treatment is about 30 microns and that the morphology is characterized by a recrystallized structure. After thermal treatment for 24 h in an air furnace, the grain size increased to about 55 microns, and after thermal treatment for 24 h in a vacuum furnace, it increased to about 112.5 microns.
